# Lung Cancer Cells-Controlled Dkk-1 Production in Brain Metastatic Cascade Drive Microglia to Acquire a Pro-tumorigenic Phenotype

**DOI:** 10.3389/fcell.2020.591405

**Published:** 2020-12-15

**Authors:** Dong-Xue Gan, Yi-Bei Wang, Ming-Yang He, Zi-Yang Chen, Xiao-Xue Qin, Zi-Wei Miao, Yu-Hua Chen, Bo Li

**Affiliations:** Key Laboratory of Medical Cell Biology, Ministry of Education, Department of Developmental Cell Biology, School of Life Sciences, China Medical University, Shenyang, China

**Keywords:** Dkk-1, lung cancer, exosomes, brain endothelial cells, microglia, neurovascular units, brain metastasis

## Abstract

**Objectives:**

Organotropism is primarily determined by tumor-derived exosomes. To date, the role of lung cancer cells-derived exosomes underlying the pre-metastatic niche formation is unclear.

**Materials and Methods:**

The animal models of retro-orbital and intra-ventricular injection were constructed to administrate lung cancer cells-derived exosomes. Cytokine array was used to screen the cytokines released from brain endothelium after internalization of lung cancer cells-derived exosomes. The cellular co-culture system was established to mimic microglia-vascular niche contained lung cancer cells-derived exosomes. The levels of Dkk-1 and the activities of microglia were analyzed by qRT-PCR, western blot and immunofluorescence. *In vivo* selections of highly brain metastatic cells were performed to analyze the direct interaction of lung cancer cells with microglia.

**Results:**

Animal studies demonstrated that there was a suppressive signal transferred from brain endothelium to microglia after internalization of lung cancer cells-derived exosomes into brain endothelium, which caused an absolutely less M1 phenotypic microglia and a relatively more M2 phenotypic microglia. Further results indicated that lung cancer cells-derived exosomes induced a release of endogenous Dkk-1 from brain endothelium, which rendered microglia to acquire a pro-tumorigenic feature in pre-metastatic niche. Subsequently, the declines of Dkk-1 in metastatic lung cancer cells removed the suppression on microglia and enhanced microglial activation in metastatic niche.

**Conclusion:**

Our findings shed a new light on the synergistic reaction of the different cells in “neurovascular units” toward the metastatic messages from lung cancer cells and provided a potential therapeutic pathway for lung cancer metastasis to brain.

## Introduction

Brain metastasis (BrM) is known as the mortal complication of tumor. Traditional radiation and surgery are insufficient for the control of BrM, and 80% patients with symptomatic BrM have survived for less than 1 year (Nayak et al., 2012). The different cancer types and subtypes display preferentially metastasize to brain, known as “organotropism to brain.” Lung cancer is the primary tumor that most commonly metastasizes to the brain. The epidemiological data indicated that about 20% of lung cancers spread to brain, which took up the highest proportion of any cancer types (Gould, 2018). However, it is still unclear why lung cancer metastasize to brain more frequently than others. Thus, a better understanding of the pathogenesis of lung cancer metastasis to brain is critical for the development of more effective therapies.

Many studies focused largely on identifying cell-intrinsic determinants of organotropic metastasis, including genes and chemokine receptors expressed on cancer cells. The adhesion and extracellular matrix molecules, such as integrins, tenascin and periostin, had been also shown to promote colonization of metastatic cancer cells. Among them, extracellular vesicles, especially exosomes were paid closed attention. Exosomes were nano-sized vesicles (30–150 nm diameters) enclosed by a lipid bilayer. They were secreted by most cells, which were as a mean of intercellular communication by transporting various biomolecules, including proteins, lipids, RNA, and DNA (Steinbichler et al., 2017; Gao et al., 2019; Yuzhalin and Yu, 2019). Several studies had provided enough evidence that tumor-derived exosomes could conduct “pre-metastatic niches” prior to the arrival of tumor cells (Lobb et al., 2017). Lyden’s study revealed that exosomes derived from organotropic metastatic cancer cells could be preferentially up-taken by specific host organ cells to create the pre-metastatic niche (Hoshino et al., 2015). Exosomal miR-25-3p derived from colorectal cancer cells mediated the formation of a vascular pre-metastatic niche to promote metastasis (Zeng et al., 2018). Melanoma-derived exosomes redirected the functions of stromal cells in a distinct manner, promoting the formation of an inflammatory pre-metastatic niche (Isola et al., 2016). These research results suggested that exosomes conferred to the organotropism to specific organs by unloading their cargo to residential cells in the secondary organ. The distinctive characteristic of brain metastasis was attributed to the complex components and precise structures. The association between brain microvascular cells (BMECs), pericytes, astrocytes, microglia and neurons forms functional “neurovascular units” (NVU), which together maintained the brain homeostasis (Hosford and Gourine, 2019). Recent studies highlighted the importance of NVU in brain metastasis (Phillips et al., 2016; McConnell et al., 2017; Prakash et al., 2019). Among cells of the NVU, BMECs were active in immediately responding to extracerebral tumor exosomes and continuously associating with invading tumor cells. The BMECs could secrete VEGF-A after uptook exosomes from leukemia blasts, leading to sequential BBB disruption and central nervous system (CNS) invasion (Kinjyo et al., 2019). Exosomal miR-105 from metastatic MDA-MB-231 breast cancer cells could target ZO-1, a tight junction protein, in the BMECs, which destroyed the integrity of brain endothelial monolayers and promoted brain metastasis in the animal model (Zhou et al., 2014). More interesting, the exosomes isolated from the brain-seeking subline of breast cancer cells traveled exclusively to the brain after injection retro-orbitally into mice, and 98% exosmoes harbored to BMECs (Hoshino et al., 2015). Our previous study had also shown that BMECs-derived exosomes could facilitate small cell lung cancer cells (SCLCs) to evade death signals and colonize into brain (Xu et al., 2019). These findings highlighted a possibility that BMECs might firstly receive the messages of organotropism in tumor-derived exosomes, and transferred this information to other types of cells in the NVU. However, the exact roles of lung cancer cell-derived exosomes underlying the metastatic niche formation were far from being understood.

Here, we determined the role of Dickkopf-1 (Dkk-1) initially released from the BMECs after internalization of lung cancer cells exosomes, in the formation of metastatic niche in brain. Dkk-1, a member of Dickkopf proteins family, had most extensively been characterized as an inhibitor of the canonical Wnt/β-catenin pathway. It competitively bound to the Wnt co-receptors LRP5/6, leading to the β-catenin complex degradation (Huang et al., 2018; Baetta and Banfi, 2019). Several studies had inferred the role of Dkk-1 in various malignancies. The elevated levels of Dkk-1 were correlated with a poor prognosis in patients suffering from multiple myeloma, prostate cancer, hepatocellular carcinoma and non-small cell lung cancer (NSCLC) (Politou et al., 2006; Yang et al., 2013; Dong et al., 2014; Rachner et al., 2014). Here, we reported that lung cancer cell derived-exosomes controlled the activity of microglia in pre-metastatic niche by inducing the release of Dkk-1 from the BMECs. Subsequently, the decline of Dkk-1 in the metastatic lung cancer cells would strengthen the extent of microglia activation and infiltration into in tumor mass.

## Materials and Methods

### Cell Lines and Regents

The detailed information was given in Supplemental Experimental Procedures.

### Animal Experiments

The animals used in this experiment were C57BL/6 mice, 6–8 weeks, provided by the Laboratory Animal Department of China Medical University. Mice were fee at the SPF facilities of China Medical University. All experiments involving animals were approved by the Animal Care and Use Committee of China Medical University. Firstly, mice were anesthetized by inhalation of isoflurane and inoculated via retro-orbital injection with 200 μl PBS containing 10 μg LLC exosomes every other day according to the instruction described previously (Yardeni et al., 2011). For intra-ventricular injection, a mini-osmotic pump was implanted into the brain to infuse LLC exosomes intracerebroventricularly (Hashimoto et al., 2002). After that, the delivery of 10 μg LLC exosomes was carried out every other day by retro-orbital vein and intra-ventricular injection, respectively. For *in vivo* administration of Dkk-1, mice were treated with recombinant mouse Dkk-1 at a dose of 1.25 μg in 5 μl PBS via intra-ventricular injection; the control mice received vehicle alone. Secondly, the brain metastatic cell populations from Lewis lung cancer (LLC) cells were obtained by consecutive rounds of *in vivo* selection in C57BL/6 mice. For orthotopic brain injections, the mice were anesthetized with halothane (induction 5% and maintenance 1%) and fixed to the stereo tactical frame. A midline incision was made on the scalp and a small hole was drilled onto the skull at bregma, 1 mm anteroposterior and + 1.8 mm mediolateral. A 2.5 μl Hamilton syringe with a 30-gauge needle was used to inject 10^5^ LLC cells in 2.5 μl sterile Hanks’ buffered salt solution into the brain at the depth of 1.5 mm.

### Patients and Specimens

All patients who attended Shengjing Hospital of China Medical University (CMU) from 2018 to 2019 were initially diagnosed with lung cancer. All experimental protocols were approved by the Ethical Review Board of China Medical University, and were performed in accordance with the committee guidelines. The written informed consents were obtained from all patients. Firstly, nine sets of lung cancer specimens were collected from the lung cancer patients without brain metastasis, including the primary tumor sites (T), precancerous lesions (P) (< 0.5 cm) and neighboring normal tissues (N) (< 1 cm, > 0.5 cm) in lung. The nine patients were diagnosed with adenocarcinoma (*n* = 3), squamous cell carcinoma (*n* = 2) and small cell lung cancer (*n* = 4), respectively. Meanwhile, the brain metastasis tissue samples were collected from another 10 lung cancer patients, who were undergoing craniotomy for brain tumor resection. Additionally, for isolation of circulating exosomes, the serum samples were obtained from 20 lung cancer patients without brain metastasis, including six patients with squamous cell carcinoma, eight patients with adenocarcinoma, and six patients with small cell lung cancer. Six serum samples of normal human were as the controls. All groups had a concordance with age, clinical stage, treatment regimen, and collection time. All samples were obtained from patients who had not received preoperative neo-adjuvant chemotherapy or radiation therapy. In addition, 31 primary SCLC specimens and 16 SCLC brain metastatic samples were from our previous study (Xu et al., 2019), and were re-examined for the immunohistochemistry (IHC)-based expression of Dkk-1.

### Isolation and Characterization of Lung Cancer Cells-Derived Exosomes

The isolation of exosomes derived from lung cancer cells were performed by the serial ultracentrifugation as described in our previous paper (Xu et al., 2019). The circulating exosomes from lung cancer patients were purified using the ExoQuick precipitation solution (System Biosciences, Ozyme, France) according to the manufacturer’s instructions. The purified exosomes were verified by western blot to detect the presence of the endosomal markers CD63 and CD9. The structures of exosomes were observed by transmission electron microscopy. Exosomes sizes and particle number were analyzed using the Zetasizer Nano ZS90 system (Malvern Instruments, United Kingdom).

### Immunofluorescence for Brain Slices

To harvest the brain tissues, mice were anesthetized with 1% pentobarbital sodium and transcardially perfused with PBS, followed by 4% paraformaldehyde. After that, the brains were removed and post-fixed in 4% paraformaldehyde overnight, followed by 30% sucrose overnight. The 100 μm coronal brain slices were made using vibratome (Leica, Bensheim, Germany). The serial brain slices were blocked with blocking solution for 1 h and incubated with the mixture of rat anti-CD31 antibody (1:50) and goat anti-Iba1 antibody (1:200) overnight at 4°C. Secondary antibodies, Alexa Fluor 488, anti-rat and Alexa Fluor 555, anti-goat were used for visualization by the scanning laser confocal microscope. Nuclei were visualized using 4′, 6-diamidino-2-phenylindole (DAPI, Thermo Fisher Scientific). For each brain slice, randomly selected five fields were imaged and analyzed the average number of Iba-1-positive microglia per field using the ZEN Software (Carl Zeiss). The serial sections were made every 2 mm downward from the basal plane (surface of the brain slice, 0 mm) to the bottom of the slice to image the distribution of microglia at the different depth.

### Cytokine Array

10^5^ human brain microvascular endothelial cells (HBMECs) were seeded into a 24-well insert with a membrane pore size of 0.4 μm (Transwell, Corning Costar). After incubation for 24 h, the media containing 10% FBS depleted of bovine exosomes and A549 exosomes (10 μg/ml) were added to the upper chamber. After co-culturing for 12 h, the media in the upper and lower chambers were separately collected. The analysis of cytokine array was conducted using Proteome Profiler Human XL Cytokine Array Kit (R&D) according to the manufacturer’s instructions.

### The Cellular Co-culture System *in vitro*

A cellular co-culture system was developed according to the method as described preveriously. Briefly, 10^5^ bEnd.3 cells (mouse brain microvascular endothelial cells, mBMECs) were firstly seeded placed on the upper chamber of 24-transwell polycarbonate membrane with pore sizes of 0.4 μm (Corning Costar Corp., Cambridge, MA). Meanwhile, 10^5^ LLC cells were seeded on the other 24-well plates. After co-culturing for overnight, the transwells cultured with bEnd.3 cells were inserted into the 24-well plates with LLC cells. The indicated intervention experiments were carried out as shown in the presented study.

### Cells Treatment

For exosomes uptake assay, 10^5^ BMECs (HBMECs and MBMECs) were plated on the 24-well and incubation for overnight. Then, after changing the media containing 10% FBS depleted of bovine exosomes, the exosomes derived from lung cancer cells were added and incubated for 24 h. The BMECs and co-cultured medium were collected and subjected to quantitative real-time PCR (qRT-PCR) and enzyme-linked immunosorbent assay (ELISA), respectively. For microglia polarization, the BV2 cells were pretreated with 100 ng/ml LPS and 10 ng/ml mouse recombinant IL-4 for 24 h, respectively. For the neutralization experiment, 20 μg/ml anti-Dkk-1 antibodies were added into the upper chamber of co-cultured system. IgG isotype was as the control. Likewise, mouse recombinant Dkk-1 (50 ng/ml) was applied for a rescue experiment, and bovine serum albumin (BSA) was as the control. To knock down the level of Dkk-1 in mBMECs, Dkk-1 small interfering RNA (siRNA) (Dkk-1 siRNA: 5′-GAACAAGUACCAGACUCUUTT-3′; was used as mouse Dkk-1 (NM_012242) target sequences. Non-silencing siRNA (5′-UUCUCCGAACGUGUCACGU-3′) was used as the negative control. The MBMECs were transiently transfected with siRNA using Dharma FECT siRNA Transfection Reagents (Dharmacon, Lafayette, CO) and the levels of Dkk-1 were measured at the indicated time points by western blot.

### Quantitative Real-Time PCR (qRT-PCR)

Total RNA was isolated using TRIzol reagent (Invitrogen, Thermo Fisher Scientific Inc., Waltham, MA) according with manufacturer’s instructions. The quality and quantity of purified RNA were identified a NanoDrop UV-visible spectrophotometer. The cDNA was synthesized and amplified using a PrimeScrip RT Reagent Perfect Real Time Kit and TaqMan Premix Ex Taq Perfect real-time kit (Takara Bio, Tokyo, Japan) according to the manufacturer’s protocols. The qRT-PCR was conducted on ABI PRISM 7500HT Sequence Detection System (Applied Biosystems, Thermo Fisher Scientific Inc., Waltham, MA). The sequences of primers were listed in Supplementary Table 1. The relative levels of target genes were normalized to GAPDH by 2^–ΔΔCt^ method.

### Western Blot

The mice brain tissues and cells were sonicated in cold lysis buffer (Beyotime Institute of Biotechnology, China). The lysate was centrifuged at 12,000 g for 10 min at 4°C. Then, the supernatant fraction was collected and the concentrations of total proteins were detected by the BCA protein assay reagent kit (Pierce Chemical Co., Rockford, IL). The primary antibodies were as follows: rabbit anti Dkk-1 (1: 800); rabbit anti β-tubulin (1: 1,000); rabbit anti-CD63 and CD9 (1: 1,000); rabbit anti IL-1β and arginase-1 (1: 1,000). The secondary antibodies were anti-rabbit IgG, HRP-linked antibody (1:10,000). Protein bands were visualized using chemiluminiscent reagents (Pierce) and were analyzed using Alphamanger 3400 software (Alpha Innotech Corporation, San Leandro, United States).

### Statistical Analysis

All analysis was carried out by GraphPad Prism 8.0 software. A two-tailed Student’s *t*-test was applied for the statistical comparison of two groups, and a one-way ANOVA with Tukey test was used for multiple groups. The data were presented as the mean ± SD, and *P* < 0.05 was considered statistically significant.

## Results

### The Brain Endothelia Cells Delivered an Inhibitory Signal to Microglia After Uptake of Lung Cancer Cells-Derived Exosomes

**FIGURE 1:**
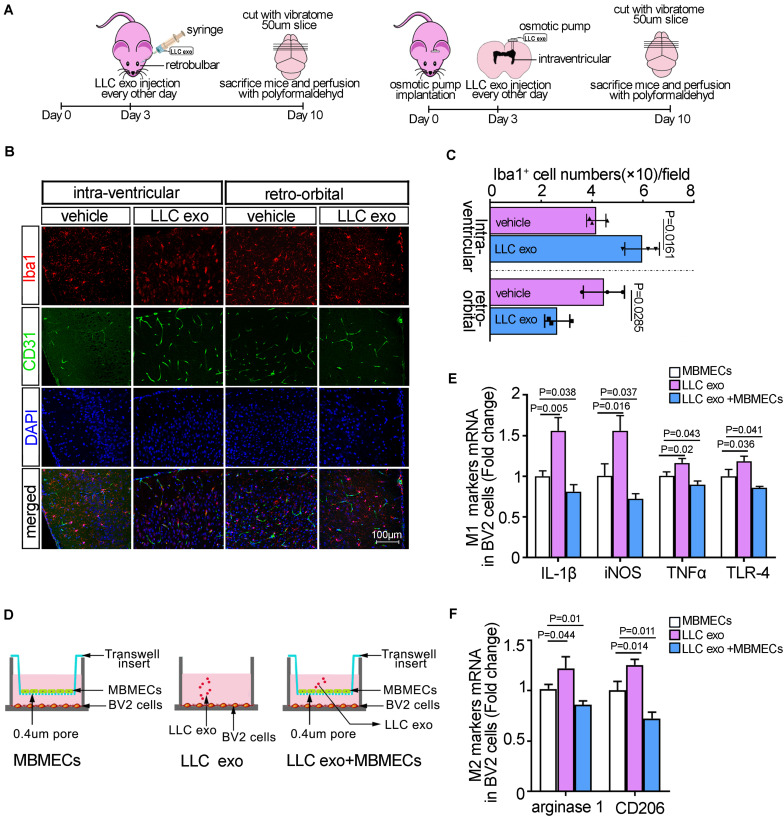
The uptake of lung cancer cells-derived exosomes by brain endothelia cells offered an inhibitor signal to microglia. **(A)** Schematic illustration of the methods of LLC exosomes administration to C57 mice. **(B)** Representative immunofluorescence staining for Iba1 and CD31 from cerebral cortex regions of the injected side after retro-orbital injection and intra-ventricular injection, respectively. Scale bar, 100 μm. **(C)** Quantitative analysis for the numbers of Iba-1-positive microglia cells. The PBS alone was as the vehicle. The histograms represented the average numbers of Iba-1-positive microglia cells from the total 6 random visual fields in three independent experiments. **(D)** The schemes of the *in vitro* cellular co-cultured experiment and the methods of LLC cell-derived exosomes treatments. **(E)** The qRT-PCR analysis of M1 markers (IL-1β, iNOS, TNFα, and TLR-4) in BV2 cells in the presence or absence of LLC cells-derived exosomes in cellular co-cultured system. The results were presented as mean ± SD of three independent experiments. **(F)** The qRT-PCR analysis of M2 markers (arginase-1 and CD206) in BV2 cells in the presence or absence of LLC cells-derived exosomes in cellular co-cultured system. The results were presented as mean ± SD of three independent experiments.

Although multiple studies had demonstrated that the residential cells in brain could be instigated by tumor-derived exosomes and participate in brain metastasis, the findings concerning the role of microglial in the progress had been limited; especially, there were no more *in vivo* experimental studies on microglial responses in pre-metastatic niche. Here, to ascertain how lung cancer cells-derived exosomes affected the function of microglia, we developed an experimental model of retro-orbital injection of mice and osmotic pump implantation for intra-ventricular injection according to the instructions as described elsewhere (Figure 1A; Hashimoto et al., 2002; Yardeni et al., 2011). Firstly, the exosomes derived from LLC cells were purified from the conditioned media by ultracentrifugation and the characteristics of exosomes were identified by Zetasizer Nano analyzer, transmission electron microscopy, western blot and flow cytometry analysis with anti-CD63 magnetic beads, respectively. The results showed a spherical, membrane encapsulated particle and the nano-size of approximately between 30 and 100 nm and the expressions of exosomal markers, CD9 and CD 63 (Supplementary Figure 1). To determine whether LLC-derived exosomes could be incorporated by BMECs, we retro-orbitally injected 10 μg of red fluorescent labeled-exosomes into C57BL/6 mice (Supplementary Figure 2A). Forty eight hours after inoculation, the distribution of exosomes in brain was visualized by laser confocal microscopy. As shown in Supplementary Figure 2B, the red fluorescently labeled exosomes was located around the vessels, which suggested that tumor exosomes in the peripheral vascular system could be caught by BMECs shown by CD31-positive immune-active. The results from immunofluorescence indicated that microglia in cortex was activated and took on an amoeboid morphology, that is, larger nuclei and cell bodies with shorter processes after intra-ventricular injection of exosomes. Iba1 was a specific marker for microglia, known as a representative of active microglia (Norden et al., 2016), and then the quantitative morphometric analysis showed that the numbers of Iba-1-positive microglia in the cortex of C57BL/6 mice was significantly increased compared to the vehicle group (*p* = 0.0258). However, after retro-orbital injection with exosomes, microglia showed the small cell bodies with numerous long and highly branching processes and the amounts of Iba-1-positive microglia were instead decreased (Figures 1B,C). These results suggested that the different deliveries of LLC derived-exosomes might elicit the opposite effects on microglia. After retro-orbital injection, BMECs could internalize the messages in LLC derived-exosomes and released a suppressive signal to microglia. While intra-ventricular injection provided LLC derived-exosomes a direct pathway to enter into the brain and directly induced the activation of microglia. To further confirm these results, we constructed the *in vitro* cellular co-culture model to mimic the microglia-vascular niche (Figure 1D). Because the activation of microglia was often considered polarized as M1 or M2 phenotype based on the expressions of cell membrane receptors and secreted factors (Ellert-Miklaszewska et al., 2013), the expressions of microglia phenotypic markers were measured by qRT-PCR. The direct exposure to LLC derived-exosomes led to BV2 cells activation as exhibiting by pronounced expressions of the characteristic M1-type markers (IL-1β, iNOS, TNFα, and TLR-4); and the characteristic M2-type markers, arginase-1 and CD206, compared to the BV2 cells co-cultured with mBMECs alone (as the controls). While there was a significant reduction in the levels of M1/M2 phenotypical markers when BV2 cells were co-cultured with mBMECs after uptake of LLC derived-exosomes, which seemed to be in accord with the *in vivo* results (Figures 1E,F). Furthermore, it was very interesting that the ratios of M2/M1 phenotypical markers were increased significantly after treatment with LLC exosomes, suggesting a more production of M2 microglia (Supplementary Figure 3). Taken together, our results suggested that there seem to be a suppressive message transferred from BMECs to microglia after the internalization of tumormal exosome into BMECs. Moreover, this message also caused the unbalance of M1 and M2 phenotypic microglia and the comparative enhancement of M2 microglia.

### Lung Cancer Cell-Derived Exosomes Induced Brain Endothelia Cells to Secrete Dkk-1

**FIGURE 2:**
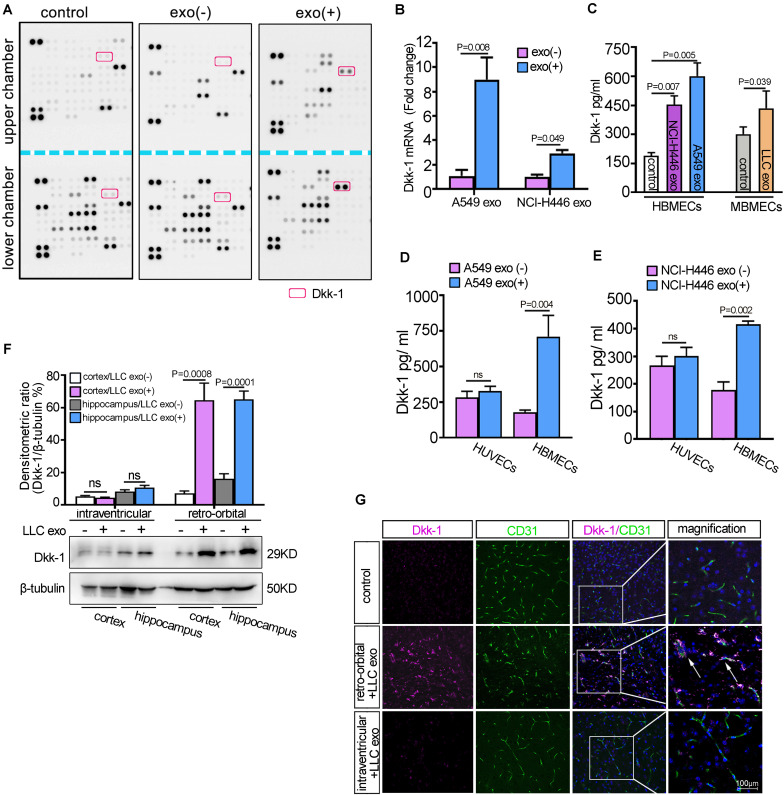
Lung cancer cells-derived exosomes induced brain endothelial cells to secret Dkk-1. **(A)** HBMECs were cultured on the membrane of Transwells in the presence or absence of A549 cells-derived exosomes and the media were collected from the upper and lower chambers, respectively. A comparative analysis of cytokine secretion was measured by Proteome Profiler Human XL Cytokine Array. **(B)** The qRT-PCR analysis of Dkk-1 in HBMECs incubated with lung cancer cells-derived exosomes. The results were presented as mean ± SD of three independent experiments. **(C)** ELISA analysis for the levels of Dkk-1 secreted by the brain endothelial cells after treatment with the indicated lung cancer cells-derived exosomes. The untreated groups were as the controls. The results were presented as mean ± SD of three independent experiments. **(D)** A comparative analysis of Dkk-1 secretion between HBMECs and HUVECs after treatment with A549 cells-derived exosomes. The results were presented as mean ± SD of three independent experiments. **(E)** A comparative analysis of Dkk-1 secretion between HBMECs and HUVECs after treatment with NCI-H446 cells-derived exosomes. The results were presented as mean ± SD of three independent experiments. **(F)** The analysis of western blots for Dkk-1 in the cerebral cortex and hippocampus regions of mice after LLC cells-derived exosomes administration by retro-orbital and intra-ventricular injection, respectively. Above: The histograms represented the average levels of Dkk-1 measured by densitometric values, normalized to the levels of β-tubulin. The results were presented as mean ± SD of three independent experiments. Below: The representative image of western blots for Dkk-1 expression was shown. **(G)** The immunofluorescence staining for the distribution of Dkk-1 in the mice brain after LLC cells-derived exosomes administration by retro-orbital and intra-ventricular injection, respectively. The inset box to right at a higher magnification. Arrows (↑) indicated the location of Dkk-1 in the brain endothelial cells. Scale bar, 100 μm.

Given that BMECs were the first responders to extra-cerebral lung cancer cell-derived exosomes, we initially evaluated the uptake of tumoral exosomes in the co-culture system according to the methods described in our previous study (Xu et al., 2019). As shown in Supplementary Figure 4, GFP-labeled HBMECs incorporated the mCherry-exosomes from A549 cells transfected with mCherry-CD63 plasmids. Next, the supernatants of HBMECs incubated with A549-derived exosomes for 24 h were collected and a profile of cytokine secretory was performed using a proteome profiler human XL cytokine array (Figure 2A). The results revealed the enrichment of Dkk-1 protein in the lower chamber compared to that of the upper chamber under A549-derived exosomes treatment. Dkk-1, a Wnt inhibitor, had been identified as a serological marker of breast cancer metastatic organotropism and inhibits lung metastasis (Zhuang et al., 2017). The levels of Dkk-1 mRNA were significantly increased in HBMECs in the presence of the indicated lung cancer cell-derived exosomes (Figure 2B, *p* = 0.008). The analyses of ELISA further confirmed an increment of extracellular levels of Dkk-1 in BMECs after treatment with lung cancer cells-derived exosomes (Figure 2C). Further results showed that there was an obvious elevation of Dkk-1 in HBMECs compared to that of HUVEC under lung cancer cell-derived exosomes treatment, suggesting that the release of Dkk-1 in the progress displayed an organtropic pattern (Figures 2D,E). In addition, HBMECs were incubated with exosomes isolated from the serum of lung patients and the supernatants were subjected to ELISA analysis. As shown in Supplementary Figure 5, the levels of Dkk-1 in HBMECs stimulated with lung cancer derived-exosomes from adenocarcinoma or SCLC were significantly higher than that of squamous cell carcinoma, which were in accordance with their potent to brain metastasis.

To further identified whether lung cancer cells-derived exosomes also induced the upregulation of Dkk-1 in BMECs *in vivo*, we constructed an experimental mice model with retro-orbital and intra-ventricular injection of 10 μg LLC exosomes as shown in Figure 1A. Then, the samples of cerebral cortex and hippocampus from the injected side of the brains were collected and the expressions of Dkk-1 were detected by western blot. As shown in Figure 2F, the expression of Dkk-1 in the groups of retro-orbital injection was higher than that of the untreated groups. However, there was no apparent change in the levels of Dkk-1 after intra-ventricular injection with LLC exosomes. Next, we sought to detect the localization of Dkk-1 protein *in vivo* after injection of LLC exosomes. The retro-orbital injection of LLC exosomes led to an increase in the numbers of Dkk-1-positive cells, and Dkk-1 was mainly localized around the cerebral vessels positively stained with CD31 (Figure 2G). Collectively, our results supported the hypothesis that BMECs might release endogenous Dkk-1 after internalization of lung cancer-derived exosomes from the peripheral vascular system and Dkk-1 might suppress the activation of microglia.

### The Release of Dkk-1 From Brain Endothelial Cells Contributed to the Suppressive Effect of Lung Cancer Cells-Derived Exosomes on Microglia

**FIGURE 3:**
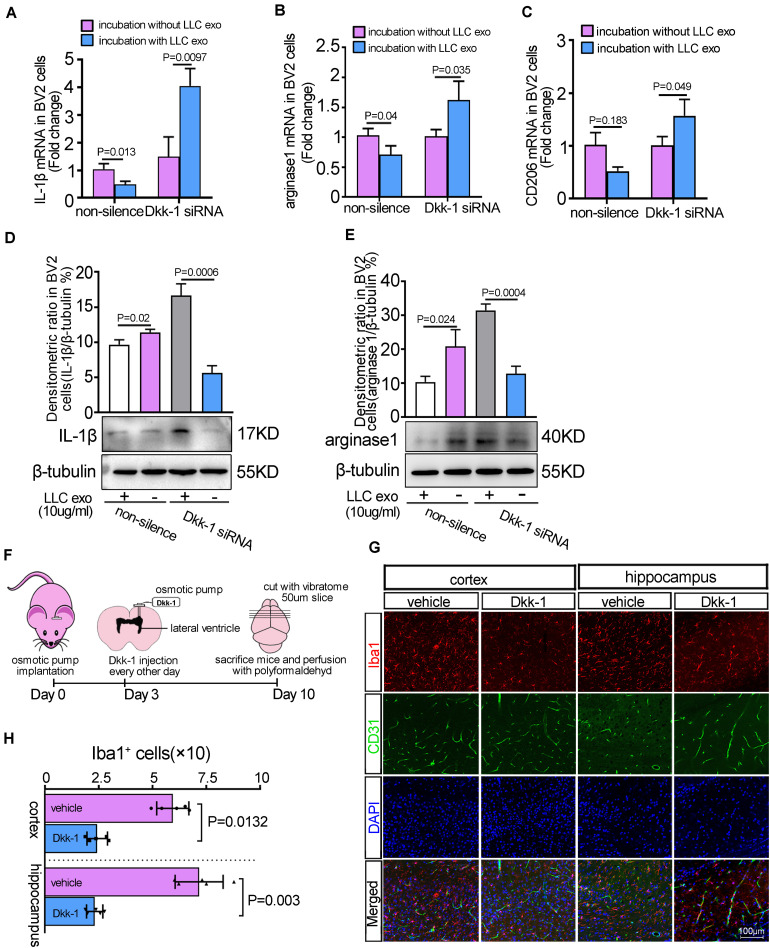
LLC cells-derived exosomes-induced the release of Dkk-1 from the brain endothelium inhibited the phenotypic polarization of microglia. The brain endothelia cells (bEnd.3 cells) were transfected with Dkk-1 siRNA to knocked down the expression of Dkk-1, and co-cultured with BV2 cells in the presence or absence of LLC cells-derived exosomes. The qRT-PCR analyses for M1 marker, IL-1β **(A)** and M2 Markers, arginase-1 **(B),** and CD206 **(C)** were shown. Results were presented as mean ± SD of three independent experiments. The quantitative analysis of western blots for M1 marker, IL-1β **(D)** and M2 marker, arginase-1 **(E)** were shown. Above: The histogram represented the average levels of target proteins measured by densitometric values, normalized to the levels of β-tubulin. Results were presented as mean ± SD of three independent experiments. Below: The representative images of western blots for target proteins were shown. **(F)** Schematic illustration of the methods of Dkk-1 administration to mice. **(G)** The immunofluorescence staining for Iba1 and CD31 in the injected sides of cerebral cortex and hippocampus after intra-ventricular injection of Dkk-1. Scale bar, 100 μm. **(H)** The quantitative analysis for the numbers of Iba-1-positive microglia cells. The PBS alone was as the vehicle. The histograms represented the average numbers of Iba-1-positive microglia cells from the total 6 random visual fields in three independent experiments.

To further demonstrate that Dkk-1 secreted by BMECs contributed to the suppressive effect of lung cancer cells-derived exosomes on microglia, we used RNAi to knock down the levels of Dkk-1 in MBMECs (bEnd.3 cells). A cellular co-culture system was developed according to the method as shown in Supplementary Figure 6. The addition of LLC exosomes could down-regulate the expression of representative M1and M2 signature genes, IL-1β, arginase-1 and CD206 in BV2 cells, while knock down of Dkk-1 eliminated these effects, as evidenced with the significant up-regulation of M1 and M2 markers in BV2 cells (Figures 3A–C). Meanwhile, the proportions of M2/M1 microglia had fallen when Dkk-1 was depleted in the BMECs (Supplementary Figure 7), which suggested that Dkk-1 released by the BMECs was required for a more production of M2 microglia in brain pre-metastatic niche. Further results from western blot also displayed the similar alterations in the levels of IL-1β (Figure 3D) and arginase-1 (Figure 3E). Next, the effects of Dkk-1 on microglial *in vivo* were further analyzed through delivering recombinant Dkk-1 protein into mice brain using osmotic pump implantation (Figure 3F). The brain slices were subjected to double immunofluorescent staining against microglia marker Iba-1 and endothelial marker CD31. The results showed that Dkk-1 treatment triggered a significant reduction in the numbers of Iba-1-positive cells in cerebral cortex and hippocampus (Figures 3G,H). However, the delivery of Dkk-1 proteins had no effect on the cerebrovascular density shown by CD31-positive cells. These results suggested that lung cancer cell derived-exosomes could indirectly modulate the activity of microglia via directly inducing the secretion of Dkk-1 in BMECs.

### Dkk-1 Hindered the Polarization of Quiescent Microglia and Induced the M1 to M2 Phenotypic Conversion of Microglia *in vitro*

**FIGURE 4:**
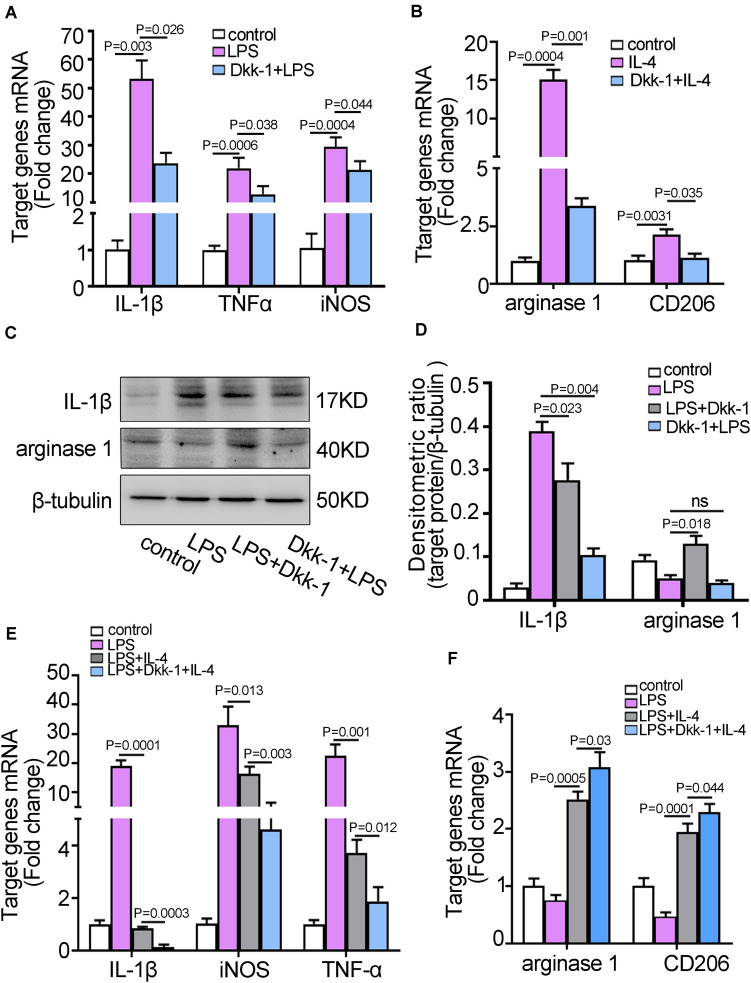
Dkk-1 suppressed M1/M2 microglia activation and stimulates M1 to M2 phenotypic conversion of microglia. **(A)** The qRT-PCR analyses for M1 markers, IL-1β, TNF-α, and iNOS, in BV2 cells pretreated with or without recombinant Dkk-1 proteins followed by LPS stimulation. The BV2 cells alone were as the control. **(B)** The qRT-PCR analyses for M2 markers, arginase-1 and CD206, in BV2 cells pretreated with or without recombinant Dkk-1 protein followed by IL-4 stimulation. The BV2 cells alone were as the controls. **(C)** The BV2 cells were stimulated with LPS prior to treatment with recombinant Dkk-1 proteins. Alternatively, The BV2 cells were pretreated with recombinant Dkk-1 proteins following exposure to LPS stimulation. The analyses of western blot for IL-1β and arginase-1 in the indicated BV2 microglia cells were shown. **(D)** The quantitative analysis of western blots for IL-1β and arginase-1 were shown. The histogram represented the average levels of target proteins measured by densitometric values using Image J software, normalized to the levels of β-tubulin. The results were presented as mean ± SD of three independent experiments. **(E,F)** The BV2 cells were pretreated with LPS to be M1 phenotypic microglia. Treatment of LPS-stimulated BV2 cells with only IL-4, or combination with recombinant Dkk-1 protein and IL-4. After 24 h, the levels of M1 markers **(E)** and M2 markers **(F)** were examined by qRT-PCR. All results were presented as mean ± SD of three independent experiments.

Microglia could be differentiated into classical (M1) or alternative (M2) phenotype under microenvironment stimulus. M1 cells were activated by lipopolysaccharide (LPS), and M2 cells were activated by type II cytokines such as IL-4 (Ellert-Miklaszewska et al., 2013; Ghosh et al., 2016). To further elucidate the effect of Dkk-1 on microglia activity, we firstly performed the inhibition experiment in co-cultured system using GW4869, which inhibited the secretion of lung cancer cell derived-exosomes. The results indicated that IL-1β expressions, a M1-specific marker, were gradually increased in the presence of GW4869, whereas the levels of M2-specific markers, arginase-1 and CD206, were decreased (Supplementary Figure 8). These results suggested that inhibition of exosomes secreted from LLC cells led to BV2 cells in co-cultured system maintained the M1 phenotypic microglia. Conversely, the appearance of lung cancer cell-derived exosomes might induce a shift of M1 to M2 phenotypic microglia. After that, BV2 cells were pretreated with Dkk-1 and then activated with LPS. The induction of M1 phenotype was shown by the levels of the characteristic markers, IL-1β, TNF-α and iNOS measured by qRT-PCR. As shown in Figure 4A, the expressions of M1-specific cytokines in LPS stimulated group were enhanced while the levels of these cytokines were significantly suppressed in Dkk-1 + LPS group (*p* = 0.026). Similarly, the production of M2 phenotype markers, arginase-1 (*p* = 0.001) and CD206 (*p* = 0.035), was also significantly suppressed in the Dkk-1 + IL-4 group when compared to IL-4 group (Figure 4B). Likewise, the results from western blot for IL-1β and arginase-1 also exhibited that Dkk-1 pretreatment inhibited LPS-induced the polarization of M1 microglia. When BV2 cells were activated with LPS and then treated with Dkk-1, an unexpected elevation of arginase-1 concomitant with a reduction of IL-1β was indicative of a phenotype conversion of M1 (IL-1β^+^) to M2 microglia (arginase-1^+^) (Figures 4C,D). Next, we further investigated whether Dkk-1 was involved in the phenotype conversion from M1 to M2 microglia. As shown in Figure 4E, LPS exposure alone induced the upregulation of M1-specific cytokines, IL-1β, TNF-α, and iNOS, which were significantly down-regulated after IL-4 addition. Meanwhile, the elevated expressions of M2 phenotypic markers, arginase-1 and CD206, were shown in the LPS + IL-4 group (Figure 4F). These results indicated that LPS-activated microglia converted to M2 microglia under IL-4 stimulation. Interestingly, in LPS-activated microglia, concurrent application of Dkk-1 and IL-4 dramatically led to a reduction of M1 markers and an increment of M2 markers compared to combination of LPS and IL-4, suggesting that there was a synergistic effect of Dkk-1 and IL-4 for a transition to M2 phenotype (Figures 4E,F). Our results suggested that Dkk-1 could impair the sensitivity and reactiveness of microglia to inflammatory stimulus, but conferred to a synergism signal to facilitate the phenotypic conversion from M1 to M2 microglia. Therefore, an absolutely less M1 microglia and a relatively more M2 microglia might contribute to the initial phase of pre-metastatic niche with an immunosuppressive state. To further demonstrate the effect of lung cancer cells-derived exosomes on lung cancer metastasis to brain, we analyzed the ability of lung cancer cells transendothelial migration using Transwell system with the appearance of microglia. As shown in Supplementary Figure 9, pretreatment with LLC cells-derived exosomes to brain endothelium co-cultured with BV2 cells promoted the transendothelial migration of LLC cells, while knockdown of Dkk-1 in brain endothelium abolished the effects. Although this was not an *in vivo* experiment to demonstrate that the changes of the pre-metastatic niche, or Dkk-1 manipulation, increases brain metastasis, these results at least partly indicated the release of Dkk-1 from brain endothelium after uptake of lung cancer cells-derived exosomes contributed to the transendothelial migration of lung cancer cells. After that, to ascertain how Dkk-1 induced M1 to M2 microglia switches, we detected the related pathway involved in the phenotype of microglia, such as Wnt/β-catenin and AMPK. As shown in Supplementary Figure 10, the addition of LLC exosomes into brain endothelial cells could induce the AMPK activation in BV2 cells of co-cultured system, but knockdown of Dkk-1 abolished these effects. However, there were almost no changes in the levels of β-catenin, a key factor of Wnt pathway. These results suggested that AMPK signal might be correlation with the role of Dkk-1 in microglia phenotype. Then we further examined the activation of AMPK in the process of Dkk-1-induced M1 to M2 microglia switches. When LPS-activated microglia converted to M2 microglia under IL-4 stimulation, the phosphorylation of AMPK was obviously increased. Then, we substituted mouse recombinant Dkk-1 protein for IL-4 and obtained the similar results. Moreover, there was no effect on IL-4-activated microglia after treatment with Dkk-1. However, there were almost no changes in the levels of β-catenin. Taken together, these results suggested that AMPK signal pathway might be involved in Dkk-1-mediated M1 to M2 microglia switches.

### The Levels of Dkk-1 in Lung Cancer Cells Became Less After Colonization Into Brain

**FIGURE 5:**
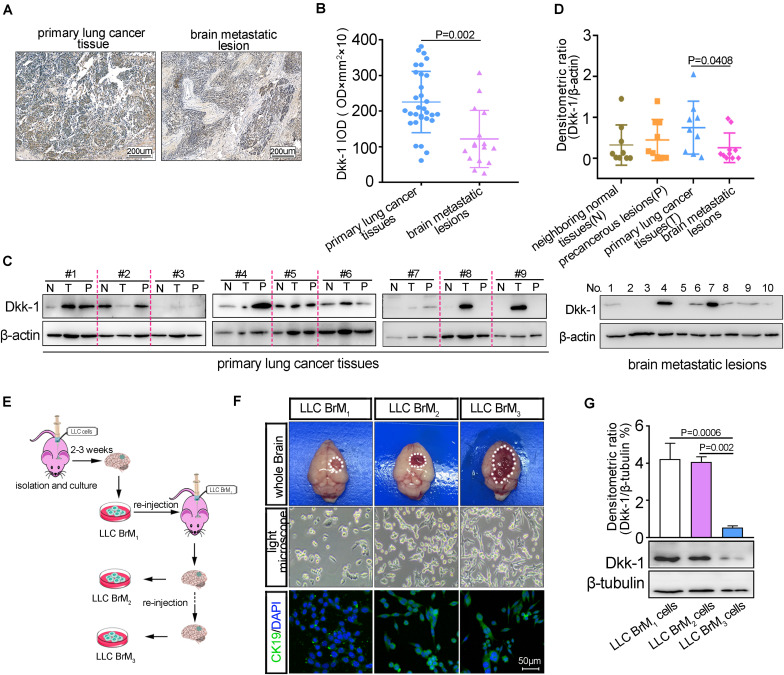
The expressions of Dkk-1 were inversely correlated to lung cancer metastasis to brain. **(A)** The representative image for immunohistochemical staining of Dkk-1 in primary lung cancer tissue and the unmatched brain metastatic lesion. Scale bar, 200 μm. **(B)** The statistical analysis of immunohistochemical staining for Dkk-1 in the primary lung cancers (*n* = 31) and unmatched brain metastatic lesions (*n* = 16). The staining intensity of Dkk-1 was quantified by average integrated optical density (IOD) values from 6 random visual fields per tissue section. **(C)** Left: the analysis of western blots for Dkk-1 in the 9 sets of samples, including the primary tumor sites (T), precancerous lesions (*P* < 0.5 cm) and neighboring normal tissues (N < 1 cm, > 0.5 cm) obtained from the same lung cancer patient. Right: the analysis of western blots for Dkk-1 in the brain metastatic lesions from the other patients with lung cancer (BrM, *n* = 10). **(D)** The quantitative analyses of densitometric values of blots (data in **C**, normalized to the levels of β-actin.) from 9 sets of samples derived from lung cancer patients and the brain metastatic lesions derived from the other lung cancer patients (BrM, *n* = 10). **(E)** Schematic illustration of *in vivo* selection of highly brain metastatic derivative lung cancer cells (LLC BrMs). **(F)** The identification of *in vivo* selection of highly brain metastatic derivative cells. Fourteen days after orthotopic brain injection of LLC BrM cells, the representative images of the tumor mass in mice brains were shown by the white circle. The brain sections were subjected to histological analyses by light microscope and immunofluorescence staining for CK19, as a marker of LLC cells. Scale bar, 50 μm. **(G)** The expressions of Dkk-1 in three rounds brain metastatic derivative cells were detected by western blot. β-actin was used as the loading control. The densities of Dkk-1 were quantified using Image J software and the results were presented as the amounts of Dkk-1 normalized against β-tubulin. A representative blot was shown in the below.

Accumulated clinical evidence had demonstrated that high levels of Dkk-1 were correlated with poor overall survival in various cancers, which suggested that Dkk-1 might be as a prognostic marker (Politou et al., 2006; Yang et al., 2013; Dong et al., 2014; Rachner et al., 2014). Having demonstrated that brain endothelium-derived Dkk-1 directly triggered microglia to promote the formation of pre-metastatic niche, we next sought to evaluate whether Dkk-1 was also involved in the progression of lung cancer metastasis to brain. Firstly, the IHC analysis displayed that there was a decline of Dkk-1 expression in the brain metastatic lesions compared to the primary lung cancer tissues (Figures 5A,B). Meanwhile, the results from western blots showed that the high expression of Dkk-1 was observed in the primary tumor tissues and the associated precancerous lesions, while the neighboring lung tissues showed the low expression of Dkk-1 (Figures 5C,D). More importantly, the decline of Dkk-1 expression had been also observed in the brain metastatic lesions compared to the primary tumor tissues in lung (Figures 5C,D). To further confirm the reduction of Dkk-1 in lung cancer cells after colonization into brain, we performed the *in vivo* selection of highly metastatic lung cancer cells as described in other study (Bos et al., 2009). Firstly, the parental LLC cells were directly injected into C57 mice brain using stereotaxic apparatus. After tumor micro-dissociation and expansion in culture, the obtained cell populations (brain metastatic derivative 1, BrM1) were re-inoculated into mice, yielding BrM2 cell populations. Then, a third round of selection *in vivo* yielded BrM3 cell populations (Figure 5E). Histological analysis showed that LLC BrM3 lesions replaced large areas of the brain parenchyma, suggesting more rapid and efficient colonization into the brain. Moreover, LLC BrM3 cells exhibited the increased activity of growth *in vitro* (Figure 5F). Next, we examined the expressions of Dkk-1 in the metastatic cell populations and found a declining trend for Dkk-1 expression along with their abilities to colonization into the brain, which were similar with the *in vivo* results (Figure 5G). Taken together, these results implied a possibility that the levels of Dkk-1 in lung cancer cells became descend after colonization into brain.

### The Reduction of Dkk-1 in Brain Metastatic Lung Cancer Cells Conferred the Activation of Microglia in Metastatic Microenvironment

**FIGURE 6:**
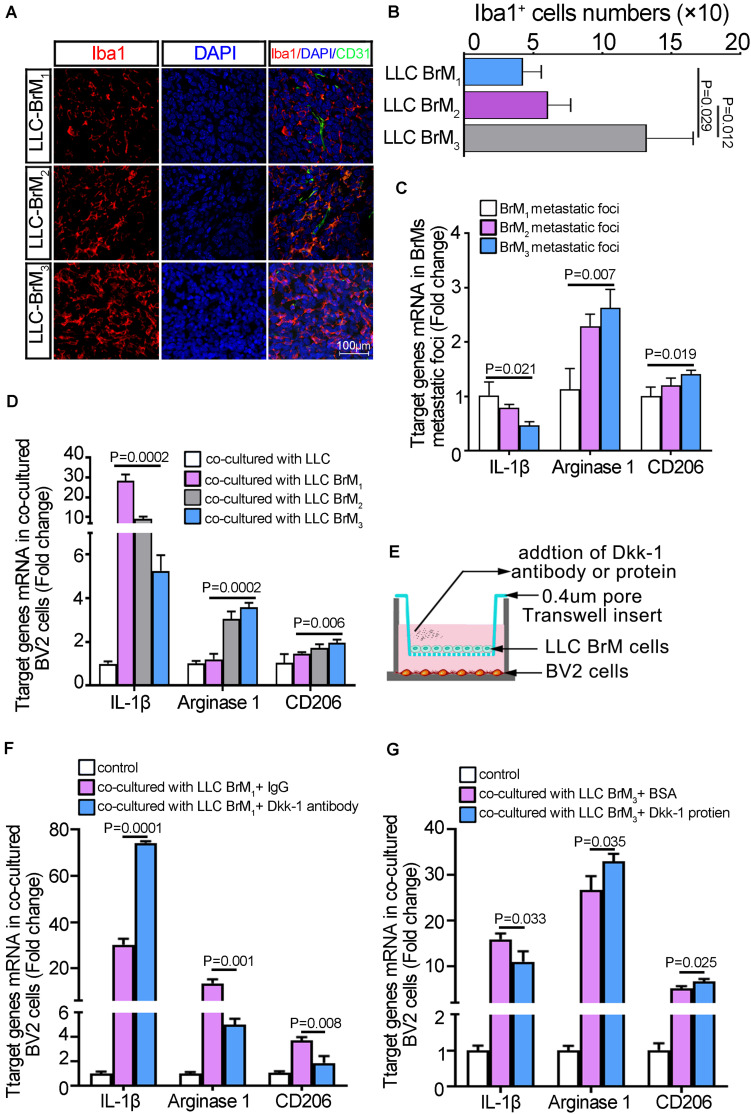
The reduction of Dkk-1 in brain metastatic lung cancer cells was conducive to the phenotypic polarization of microglia in metastatic microenvironment. **(A)** The confocal microscopy images of immunofluorescence staining for Iba1 in the brain sections of LLC BrM cells-bearing mice. Scale bar, 100 μm. **(B)** The quantitative analysis for the numbers of Iba-1-positive microglia cells in LLC BrM cells-bearing mice brain. The histograms represented the average numbers of Iba-1-positive microglia cells from the total 6 random visual fields in three independent experiments. **(C)** The qRT-PCR analyses for M1/M2-like markers in brain metastatic foci microdissected from the LLC BrMs-bearing mice. The results were presented as mean ± SD of three independent experiments. **(D)** The qRT-PCR analyses for M1/M2-like markers in BV2 cells co-cultured with LLC brain metastatic derivative cells. The results were presented as mean ± SD of three independent experiments. (E) Schematic illustration of the neutralized or rescued experiment through treatment with an anti-Dkk-1 antibody or a recombinant Dkk-1 protein, respectively. (F) The qRT-PCR analyses for M1/M2-like markers, IL-1β, arginase-1 and CD206, in BV2 cells co-cultured with LLC BrM_1_ cells as shown in **(E)**. The rabbit IgG was used as the control. The results were presented as mean ± SD of three independent experiments. **(G)** The qRT-PCR analyses for M1/M2-like markers, IL-1β, arginase-1 and CD206, in BV2 cells co-cultured with LLC BrM_3_ cells as shown in E. BSA was used as the control. The results were presented as mean ± SD of three independent experiments.

To determine the extent to which microglia was directly trigged in brain metastatic lesions, we collected the brain tissue sections after inoculation with three rounds of LLC cell populations. Confocal microscopy of immunofluorescence showed that the activated microglia cells were identified as round cells with shortened cell process and immune-reactive to Iba1. Along with the enlargement of metastatic lesions, the microglia massively was infiltrating into the metastatic foci (Figure 6A). Moreover, the amounts of Iba-1-positive microglia cells were increased dramatically in the LLC BrM3-derived lesions compared to the other groups (Figure 6B and Supplementary Figure 11). Because the expression of Iba1 only indicated the highly activated microglia in brain, we further analyzed the expression of M1/M2-like markers in brain metastatic foci micro-dissected from the LLC BrMs-bearing mice. The results showed that IL-1β expressions, a M1-specific marker, were gradually decreased from LLC BrM1 to LLC BrM3 metastatic foci, whereas the levels of M2-specific markers, arginase-1 and CD206, were on the rise (Figure 6C). Therefore, further results from the cellular co-cultured system *in vitro* confirmed that the potency of LLC cells colonization into brain was a determinant of the degree of microglia activation (Figure 6D and Supplementary Figure 12A). Therefore, it was a possibility that the decline of Dkk-1 in lung cancer cells was a result of lung cancer cells survival in the brain. To test this hypothesis, we performed the neutralized and rescued experiments through the addition of anti-Dkk-1 antibody and a recombinant Dkk-1 protein, respectively. As shown in Figure 6E, the different LLC BrMs cells were seeded on Transwell membrane of the apical chambers and BV2 cells were plated in the basal chambers. Given the levels of Dkk-1 in three BrM cell lines, we treated LLC BrM1 cells with a blocking Dkk-1-specific antibody and LLC BrM3 cells with a recombinant Dkk-1 protein, respectively. Compared to the IgG control, the blockage of Dkk-1 derived from LLC BrM1 cells could obviously lead to an increment of M1 marker and a reduction of M2 markers (Figure 6F). Instead, the application of recombinant Dkk-1 protein to LLC BrM3 cells brought about the opposite results (Figure 6G). Moreover, a shift of M1 to M2 phenotypic microglia was shown in LPS-active BV2 microglia co-cultured with the different BrMs cell populations, which was dependent on the abilities of BrMs cells colonization into the brain (Supplementary Figures 12B,C). Collectively, our results suggested that a reduction of endogenous Dkk-1 in lung cancer cells after colonization into brain would control microglia to acquire a pro-tumorigenic phenotype.

## Discussion

The microenvironment is gradually recognized as a profound determinant of tumor progression and therapeutic target. Composed of neurons, glial and protected by the BBB, the brain microenvironment is very complicated and unique. The whole microenvironment regulation of the brain metastatic cascade is pretty complicated, such as BBB transmigration, pro-apoptotic functions, astrocyte crosstalk, T lymphocytes cell responses and so on. Here, our data revealed that lung cancer cells could control the levels of Dkk1 in brain metastatic cascades, leading to the shaping of the pro-tumorigenic microenvironment in different phases: a pre-metastatic niche with insensitive microglia without tumor elements, and a micro-metastatic niche characterized by the presence of active microglia and lung cancer cells.

A key question in brain metastasis was the brain-preferential localization of certain tumor. It had been identified that tumor-derived exosome was a key regulator for the formation of the pre-metastatic niche in the secondary organs, serving as a mean of intercellular communication by transporting various biomolecules. Accumulated evidence had demonstrated that tumor-derived exosomes could educate brain-resident cells, such as brain endothelial cells, astrocytes, microglia, and neurons, favoring the disseminated tumor cells colonization into the brain (Hoshino et al., 2015; Zhang et al., 2015; Hoshide and Jandial, 2017; Kinjyo et al., 2019; Schulz et al., 2019; Xu et al., 2019). Microglia was regarded as the innate immune cell of CNS. As part of their constant surveillance, microglia executed host defense against infectious agents and neoplastic tumors in the CNS. Most *in vitro* and *in vivo* studies had mainly focused on the direct effects of tumor cell-derived exosomes on microglia, and indicated that tumor cell-derived exosomes could activate microglia to accelerate growth and invasion of metastatic tumors (Gener Lahav et al., 2019). A recent study reported that uptake of CEMIP exosomes by microglial cells in *ex vivo* brain slice induced a pro-inflammatory signature microglia (M1) to promote brain vascular remodeling and metastasis (Rodrigues et al., 2019). However, the other study showed that the exosomes derived from the XIST-knockdown breast cancer cells induced the conversion of microglia from M1 to M2 phenotype in the brain metastatic lesions (Xing et al., 2018). Moreover, 98% exosomes derived from the brain-seeking subline of breast cancer cells almost completely harbored into brain endothelium after retro-orbital injection into mice, suggesting that brain endothelial cell was an first recipient for exosomes from extra-cerebral cancer cells (Bos et al., 2009; Hoshino et al., 2015). Here, we constructed two animal models for inoculation with lung cancer cells-derived exosomes and observed the effects of tumor exosomes when they entered the brain via the different routes, retro-orbital vein and cerebral ventricle, respectively. Similarly, our results also indicated that the brain endothelia cells predominantly up-took lung cancer cells-derived exosomes *in vivo* and *in vitro*. Moreover, the retro-orbital injection of LLC-derived exosomes, mimicking the systemic exosomes released by primary tumor traveling to brain, led to a significant reduction of Iba1-positive cells in the cortex compared to the intra-ventricular injection. The cellular co-cultured experiments further indicated that a suppressive signal was outputted by brain endothelium after internalization with LLC-derived exosomes to attenuate the activity of microglia inside brain. Recently, the concept of the NVU was proposed to emphasize the unique intimate relationship between the intra-cerebral cells and the cerebral vasculature. The dynamic communication between cancer cells and NVU elements provided insights into the role of brain microenvironment in metastasis. It had been well reported that the components in NVU played the crucial roles in creating a permissive niche that driven metastatic cascade (Phillips et al., 2016; McConnell et al., 2017; Gener Lahav et al., 2019; Prakash et al., 2019; Schulz et al., 2019). Among them, brain-resident microglia was responsible for the immune privileged status of CNS. It was tempting to speculate that the initial and essential role of microglia at the pre-metastatic niche might be the resistance to the invading tumor cells. Different microglial-derived factors including proteases (e.g., Ctss, Mmp3, and Mmp9), Wnt signaling components or chemokines (e.g., Cxcl12) have been implicated in assisting tumor cells to cross the BBB and colonize the brain parenchyma. Here, our findings showed that there was an absolutely less M1 polarized microglia and a relatively more M2 polarized microglia in pre-metastatic cerebral parenchyma after the primary lung cancer cells-derived exosomes had been incorporated into the brain endothelium. Therefore, we inferred that the primary lung cancer cells-derived exosomes might transfer a suppressive signal from brain endothelium to microglia, which facilitated microglia in the pre-metastatic niche to acquire a tumor-promoting phenotype.

What was an inhibitor signal occurred in the brain endothelium after uptake of lung cancer exosomes? To ascertain the question, we performed a profile of cytokines to look for a relative factor. The Dkk-1 was identified as a potential effector based on its inhibitory effect on Wnt signaling, a pathway that was always over-activated in cancer. Dkk-1 had been widely investigated in various cancers, in which high levels of Dkk-1 were correlated with poor overall survival (Politou et al., 2006; Yang et al., 2013; Dong et al., 2014; Rachner et al., 2014). Additionally, some studies implied that Dkk-1 could serve as a modulator to alter the cancer metastasis-associated microenvironment. In the context of multiple myeloma, the Dkk-1 in the bone microenvironment contributed to the development of focal osteolytic lesions and indirectly facilitated multiple myeloma metastases to bone (Faict et al., 2018). Dkk-1 promoted vasculogenic mimicry formation by inducing NSCLC cells to acquire cancer stem-like cell characteristics (Yao et al., 2016). These findings suggested that Dkk-1displayed more pleiotropic effects in tumor development, not only transforming tumor cells themselves, but also creating a hospitable niche that allowed tumor cells survival and proliferation. A recent study demonstrated that Dkk-1 was a Janus-faced molecule, that is, Dkk-1 could promote breast-to-bone metastasis and inhibit breast-to-lung metastasis (Zhuang et al., 2017). It proposed concept that the microenvironment in the secondary organs might restrict the role of Dkk-1 in cancer metastasis. Because the arrival of lung cancer exosomes was prior to lung cancer cells colonization into brain, the release of Dkk-1 from brain endothelium after uptake of exosomes seemed to be an early event. To exclude whether tumor-derived exosomes carried Dkk-1 and transferred to brain endothelial cells, we examined the levels of Dkk-1 in the different lung cancer cells-derived exosomes and found that there was no Dkk-1 detected in lung cancer cells-derived exosomes (Supplementary Figure 13). Dkk-1 not only impaired the sensitivity and reactiveness of microglia to inflammatory stimuli, but provided a synergism signal to facilitate the phenotypic switch of microglia. Thus, an absolutely less M1 polarized microglia and a relatively more M2 polarized microglia might cause the deficits of immune response during the initial phase of pre-metastatic niche formation. It had been reported that Dkk-1 played an immune-modulatory role through direct targeting of β-catenin in myeloid-derived suppressor cells (MDSC) (D’Amico et al., 2016). Here, we revealed that Dkk-1 secreted by brain endothelium contributed to the immune suppression in pre-metastatic niche by controlling the plasticity of microglia. That is, Dkk-1 might directly hold microglia in an inactive state; meanwhile indirectly drive the existed pro-inflammatory M1-like microglia to transform into anti-inflammatory M2-like microglia. Thus, a supportive microenvironment in brain was built at the future metastatic site. A growing body of evidence had indicated that tumoral exosomes could weaken the activities of immune cells in the microenvironment, which diminished the immune surveillance (Greening et al., 2015). Given that the role of microglia was to guard, detect and respond to any insult; the insensitivity of microglia would weakened immune response in brain. For example, microglia activation was known as mandatory for the induction of T and B cells response to clean tumor cells, but loss of microglia would attenuate these effects (Yin et al., 2017). In the present study, we provided evidence to demonstrate that brain endothelium could accept the information of lung cancer cells-derived exosomes, and subsequently transfer a suppressive signal to microglia, resulting in a transition of active phenotype.

Because there was a more complicated cell-cell communication in brain, the role of NVU in the development of brain metastasis should be taken into account. To further confirm our results, we constructed the intra-cortical injection of LLC cells into mice in order to bypass the extravasation step of brain metastasis. It had been reported that even only a single tumor cell was sufficient to recruit and activate microglia (Van Hove et al., 2019). In established brain metastasis, tumor-associated microglia were the most abundant non-cancerous cell type and constituted up to 30% of the total tumor mass (Graeber et al., 2002; Watters et al., 2005). Here, the persistent re-inoculation of LLC cells enhanced their brain-tropic abilities, along with a substantial infiltration of M2-microglia into the tumor mass. However, the levels of Dkk-1 were instead decreased in LLC BrM cells with preferential colonization to brain. The analysis of clinical data was also supported that the levels of Dkk-1 was negatively correlation with the occurrence of lung cancer metastasis to brain. Further *in vitro* experiments demonstrated that brain metastatic lung cancer cells with low Dkk-1 induced microglia to obtain an anti-inflammatory property, known as a feature of brain micro-metastases. Our results were in accord with a serial previous study of brain metastasis, which had provided evidence to underline the role of Wnt signaling during cerebral colonization (Nguyen et al., 2009; Pukrop et al., 2010; Klemm et al., 2011).

**FIGURE 7:**
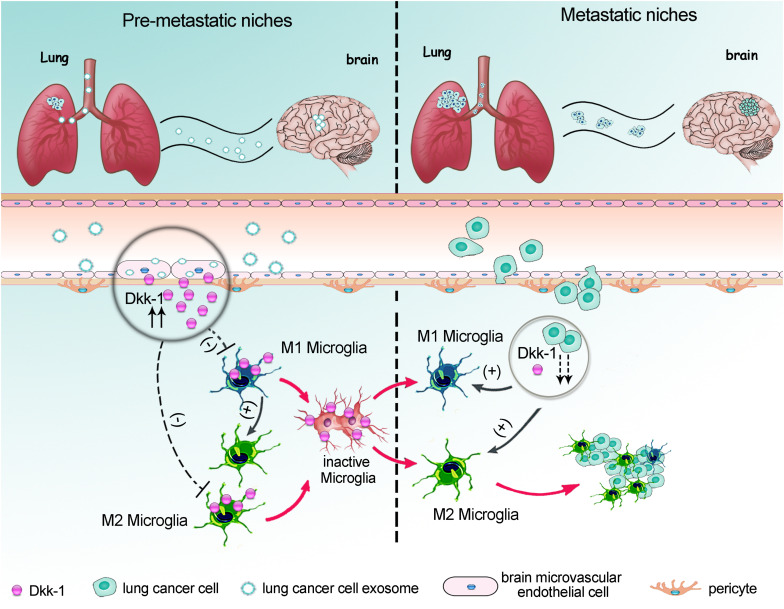
A proposed model for lung cancer cells-controlled Dkk-1 production in brain metastatic cascades driving microglia to acquire a pro-tumorigenic phenotype. As a major component of BBB, the brain microvascular endothelia cells were the primary target for lung cancer-derived exosomes. The incipient exosomes induced the release of Dkk-1 from brain endothelial cells. The Dkk-1 inside brain might directly hold microglia in an inactive state; meanwhile indirectly drive the existed M1-like microglia to convert into the pro-tumorigenic M2-like microglia. An absolutely less M1 polarized microglia and a relatively more M2 polarized microglia caused the deficits of immune response during the initial phase of pre-metastatic niche formation. When the metastatic lung cancer cells emerged into brain, the decline of Dkk-1 in metastatic lung cancer cells would facilitate microglia to acquire tumor-supportive phenotype and massively infiltration into metastatic lesion.

In summary, we proposed a model whereby as a major component of BBB, the brain microvascular endothelium was an incipient target for lung cancer-derived exosomes. The lung cancer-derived exosomes induced brain endothelial cells to secrete Dkk-1. The Dkk-1 inside brain might directly hold microglia in an inactive state; meanwhile indirectly drive the existed M1-like microglia to convert into pro-tumorigenic M2-like microglia. These changes caused the deficits of immune response during the initial phase of pre-metastatic niche formation. When the metastatic cancer cells colonized into brain, the decline of Dkk-1 would remove the limitation of immune suppression on microglia and facilitate them to acquire a tumor-supportive phenotype (Figure 7). Our findings shed a new light on the synergistic reaction of the different cells in NVU toward the metastatic messages from primary lung cancer cells, emphasizing the unique role of NVU in organotropism to brain. Therefore, the interventions that control early activation of microglia might provide a potential therapeutic pathway for lung cancer metastasis to brain.

## Data Availability Statement

The original contributions presented in the study are included in the article/Supplementary Materials, further inquiries can be directed to the corresponding author/s.

## Ethics Statement

The studies involving human participants were reviewed and approved by the Ethical Review Board of China Medical University. The patients/participants provided their written informed consent to participate in this study. The animal study was reviewed and approved by the Laboratory Animal Department of China Medical University.

## Author Contributions

BL and Z-WM designed the research and wrote the manuscript. D-XG performed the majority of experiments. Y-BW collected and analyzed the clinical experimental data and finished the revised experiments. M-YH and Z-YC assisted with the animal experiments. X-XQ and BL performed the figures and the statistical analyses. Y-HC provided the technical supports. All authors read and approved the final manuscript.

## Conflict of Interest

The authors declare that the research was conducted in the absence of any commercial or financial relationships that could be construed as a potential conflict of interest.

## Abbreviations

NVU: neurovascular units
SCLC: small cell lung cancer
NSCLC: no small cell lung cancer
LLC: Lewis lung cancer
BBB: blood-brain barrier
HBMECs: human brain microvascular endothelial cells
Dkk-1: Dickkopf-1
GFP: green fluorescent protein
CMU: China Medical University
qRT-PCR: quantitative real-time PCR
ELISA: enzyme-linked immunosorbent assay
LPS: lipopolysaccharide
BrM: brain metastatic derivative
IL-4: interleukin-4
TNFα: tumor necrosis factor alpha
TLR-4: Toll-like receptor4
IL-1β: interleukin-1 beta
iNOS: inducible nitric oxide synthase
MDSC: myeloid-derived suppressor cells.
