# Supplementary material for: Lung Cancer Cells-Controlled Dkk-1 Production in Brain Metastatic Cascade Drive Microglia to Acquire a Pro-tumorigenic Phenotype
Source: Front Cell Dev Biol. 2020 Dec 15;8:591405. doi: 10.3389/fcell.2020.591405 (PMC7769850; doi:10.3389/fcell.2020.591405)

SUPPLEMENTARY DATA

MATERIALS AND METHODS

Cell lines and regents

HBMECs were a generous gift from Dr. K.S. Kim (Department of Pediatrics, John Hopkins University School of Medicine). HBMECs were cultured in RPMI 1640 medium, supplemented with 10 % FBS (Thermo Scientific, Waltham, Massachusetts, USA) and 10 % Nu-serum (BD Biosciences, Franklin Lakes, NJ). Lewis lung cancer (LLC) cells and BV2 microglia were obtained from the Type Culture Collection of the Chinese Academy of Sciences (Shanghai, China). Human lung cancer cell lines, A549 cells, NCI-H460 cells and NCI-H446 cells were obtained from the American Type Culture Collection (ATCC) (Rockville, MD). The bEnd.3 cells, mouse microvascular endothelial cells, were also obtained from ATCC. All lung cells were cultured in RPMI 1640 supplemented with 10% FBS depleted of bovine exosomes and were maintained in 5% CO2 at 37 °C. Recombinant mouse Dkk1 protein was purchased from R&D Systems (Minneapolis, MN) and anti-Dkk1 polyclonal antibodies for neutralization experiments were obtained from Proteintech Group (Rosemont, IL). Antibodies against CD9 and CD63 for western blot were from Abcam. Anti-β-tubulin polyclonal antibodies and HRP-conjugated secondary antibodies were from Santa Cruz Biotechnology (Santa Cruz, CA). Anti-CD31 polyclonal antibody was from BD Biosciences (Billerica, MA) and anti-Iba1 antibody was obtained from Novus Biologicals (Briarwood, CO). Antibodies against IL-1β, arginase-1 and CD206 were from Cell Signaling Technology Inc. (Danvers, MA). The fluorescence labeling secondary antibodies, Alexa Fluor 488, anti-rat and Alexa-Fluor 555, anti-goat, were obtained from Thermo Fisher Scientific (Waltham, MA). Lipopolysaccharide (LPS) and mouse recombinant protein IL-4 were from Beyotime Biotechnology (Nanjing, China)

Uptake of exosomes assay in vivo

The protocol was carried out according to the methods described as the previous study [7]. Briefly, the purified exosomes were labeled with red BODIPY TR fluorophore

(Thermo Fisher Scientific) according to the manufacturer's protocol. Excess BODIPY TR was removed using Amicon filter units (Merck Millipore, Burlington, MA) and Zeba™ Spin columns (Thermo Fisher Scientific). The exosomes were diluted with PBS. To identify the entry of tumor exosomes into brain, the 6-week old naive C57BL6 mice were retro-orbitally injected 10 μg of red fluorescently labeled exosomes. 24 hrs after injection, mice were sacrificed and the 100 μm coronal brain sections were made for immunofluorescence staining with CD31 antibody to indicate the distribution of cerebral vessels. Red fluorescent-labeled LLC exosomes in brain section were imaged using the scanning laser confocal microscope (Carl Zeiss, Micrbscopy, GmbH, NY, USA).

Enzyme-linked immunosorbent assay for Dkk-1

The levels of Dkk-1 in the indicated culture supernatants were examined using the Dkk-1 ELISA assay kit ((R&D systems) according to the manufacturer's instructions.

Immunohistochemistry

All the specimens for immunohistochemical staining were fixed in 10% neutral formalin, embedded in paraffin and cut in serial sections of 5 μm. Immunohistochemical staining was performed using a peroxidase-labeled streptavidin-biotin technique. Briefly, tissue sections were deparaffinized and rehydrated. Then, sections were heated to retrieve antigenicity, and treated with 3% H_2O_2 for 10 minutes to quench endogenous peroxidase activity. After blocking non-specific binding with 10% normal goat serum, the sections were incubated overnight at 4℃ with rabbit anti-human Dkk-1 polyclonal antibody (1:100). Then, sections were treated with biotinylated donkey anti-rabbit immunoglobulins for and subsequently with a streptavidin–peroxidase conjugate (DAKO, Glostrup, Denmark). The diaminobenzidine hydrochloride (DAKO) was used to visualize the peroxidase activity. The sections were lightly counterstained with hematoxylin, mounted with Permount (Thermo Fisher Scientific), and were reviewed by two independent researchers and analyzed using the MetaMorph/DP10/BX41 image analyzing system.

Cell immunofluorescence

Brain metastatic derivative cells (5×10^3) were plated on glass coverslips over-night. Then the cells were fixed with 4% paraformaldehyde. After blocked with 5% BSA in PBS, cells were incubated with anti-cytokeratin 19 antibody (SantaCruze) at room temperature for 2 h, subsequently, mixed with anti-rabbit-IgG Alexa-Fluor-488–conjugated antibodies (1:200; Thermo Fisher Scientific) for 45 min. The slides were visualized with a confocal laser scanning microscope (Leica Microsystems).

Transendothelial migration assay

Transendothelial migration assays were performed as previously described (Li et al., 2013). As shown in supplemental Figure 9 A, after incubation for 12 hours, the top membrane surfaces were gently scraped with cotton wool to remove non-migrated cells, and the red fluorescent DiI-labeled migrated cells were counted from 10 random fields using 100 × magnifications.

SUPPLEMENTARY FIGURE LEGENS

Supplementary Fig. 1 Characterization of the lung cancer cells-derived exosomes.

A. The exosomes were purified from A549 and LLC cells cultured media by ultracentrifugation and their sizes were determined by the Zetasizer Nano ZS90 system.
B. Electron microscopic examination of A549 and LLC cells-derived exosomes. Scale bar, 100 nm.
C. Western blots of CD63 and CD9 contained in A549 and LLC cells-derived exosomes.
D. LLC cells-derived exosomes were collected and were labeled with SYTO dye, and then subjected to flow cytometry analysis with anti-CD63 magnetic beads.

Supplementary Fig. 2 Assay for Uptake of LLC cells-derived exosomes *in vivo*.

A. Schematic illustration of the methods of administration to C57 mice by retro-orbital injection
B. 10 μg of red fluorescently labeled LLC cells-derived exosomes were injected into C57 mice. 2 days after inoculation, the distribution of exosomes in brain was

visualized by confocal microscopy. Arrows (↑) indicated the LLC cells-derived exosomes. Scale bar, 100 μm.

Supplementary Fig. 3 The ratios of M2/M1 phenotypical markers in BV2 cells co-cultured with bEnd.3 cells after treatment with LLC cells-derived exosomes.

A. The analysis for the ratios of M2 (arginase 1)/M1 (IL-1β and iNOS) marker in BV2 cells in the presence or absence of LLC cells-derived exosomes in cellular co-cultured system. The results were presented as mean±SD of three independent experiments.

B. The analysis for the ratios of M2 (CD206)/M1 (IL-1β and iNOS) marker in BV2 cells in the presence or absence of LLC cells-derived exosomes in cellular co-cultured system. The results were presented as mean±SD of three independent experiments.

Supplementary Fig. 4 The uptake of the lung cancer cells derived-exosomes by HBMECs.

Left: Diagram of cellular co-culture of HBMECs transfected with pEGFP-N1 and A549 cells expressing CMV-driven mCherry-tagged CD63. Right: A representative confocal laser scanning microscope image for GFP-labeled HBMEC cells incorporating mCherry-exosomes derived from A549 cells during 4 days of co-culture. The arrows indicated mCherry-exosomes incorporated in GFP-labeled NCI-H446 cells. Scale bar, 20 μm.

Supplementary Fig. 5 ELISA analysis for the levels of Dkk-1 secreted by HBMECs after incubation with the exosomes isolated from lung cancer patients with the indicated pathological types.

Supplementary Fig. 6 Schemes of the cellular co-cultured experiment with the depletion of Dkk-1 in brain endothelial cells.

Supplementary Fig. 7 The ratios of M2/M1 phenotypical markers in BV2 cells co-cultured with Dkk-1-depleted bEnd.3 cells after treatment with LLC cells-derived exosomes.

A. The analysis for the ratio of M2 (arginase 1)/M1 (IL-1β) marker in BV2 cells co-cultured with Dkk-1-depleted bEnd.3 cells in the presence or absence of LLC

cells-derived exosomes. The results were presented as mean±SD of three independent experiments.

B. The analysis for the ratio of M2 (CD206)/M1 (IL-1β) marker in BV2 cells co-cultured with Dkk-1-depleted bEnd.3 cells in the presence or absence of LLC cells-derived exosomes. The results were presented as mean±SD of three independent experiments.

Supplementary Fig. 8 The appearance of lung cancer cell-derived exosomes might induce a shift of M1 to M2 phenotypic microglia.

The upper: The scheme of cellular co-cultured experiment of LLC BrMs cells and BV2 cells with GW4869 treatment. The below: The qRT-PCR analysis for M1/M2-like markers in BV2 cells in the co-cultured experiment with GW4869 treatment. The results were presented as mean±SD of three independent experiments.

Supplementary Fig. 9 The release of Dkk-1 from brain endothelium promoted the transendothelial migration of lung cancer cells in the presence of microglia

A. The scheme of cellular co-cultured experiment of LLC cells and mouse brain endothelium with BV2 cells under LLC cells-derived exosomes treatment.

B. Representative images of transmigrated LLC cells labeled with red fluorescent DiI dye are show.

C. Transendothelial migration assay of LLC cells in cellular co-cultured experiment.

Supplementary Fig. 10 AMPK activation was required for Dkk-1-induced M1 to M2 microglia switches.

A. The brain endothelia cells (bEnd.3 cells) were transfected with Dkk-1 siRNA to knocked down the expression of Dkk-1, and co-cultured with BV2 cells in the presence or absence of LLC cells-derived exosomes. The analyses of western blot for AMPK, the phosphorylated AMPK and β-catein in the indicated BV2 microglia cells were shown.

B. The BV2 cells were stimulated with LPS following exposure to IL-4 stimulation. Alternatively, The BV2 cells were pretreated with LPS following exposure to recombinant Dkk-1 proteins treatment. The analyses of western blot for AMPK, the phosphorylated AMPK and β-catein in the indicated BV2 microglia cells were shown.

C. The BV2 cells were stimulated with IL-4 in the presence or absence of recombinant Dkk-1 proteins. The analyses of western blot for AMPK, the phosphorylated AMPK and β-catein in the indicated BV2 microglia cells were shown.

Supplementary Fig. 11 The 2.5D intensity analyses of immunofluorescence staining for Iba1 in the brain sections of LLC BrM cells-bearing mice.

Supplementary Fig. 12 The lung cancer cells colonized into brain could induce a conversion of M1 to M2 phenotypic microglia.

A. Western blot analysis for the expressions of M1/M2-like markers in brain metastatic foci microdissected from the LLC BrMs-bearing mice.
B. The scheme of cellular co-cultured experiment of LLC BrMs cells and BV2 cells with LPS treatment.
C. The qRT-PCR analysis for M1/M2-like markers in BV2 cells in the co-cultured experiment with LPS treatment. The results were presented as mean±SD of three independent experiments.

Supplementary Fig. 13 The detection of Dkk-1 in the different lung cancer cells-derived exosomes by western blot.

Supplementary Fig.1

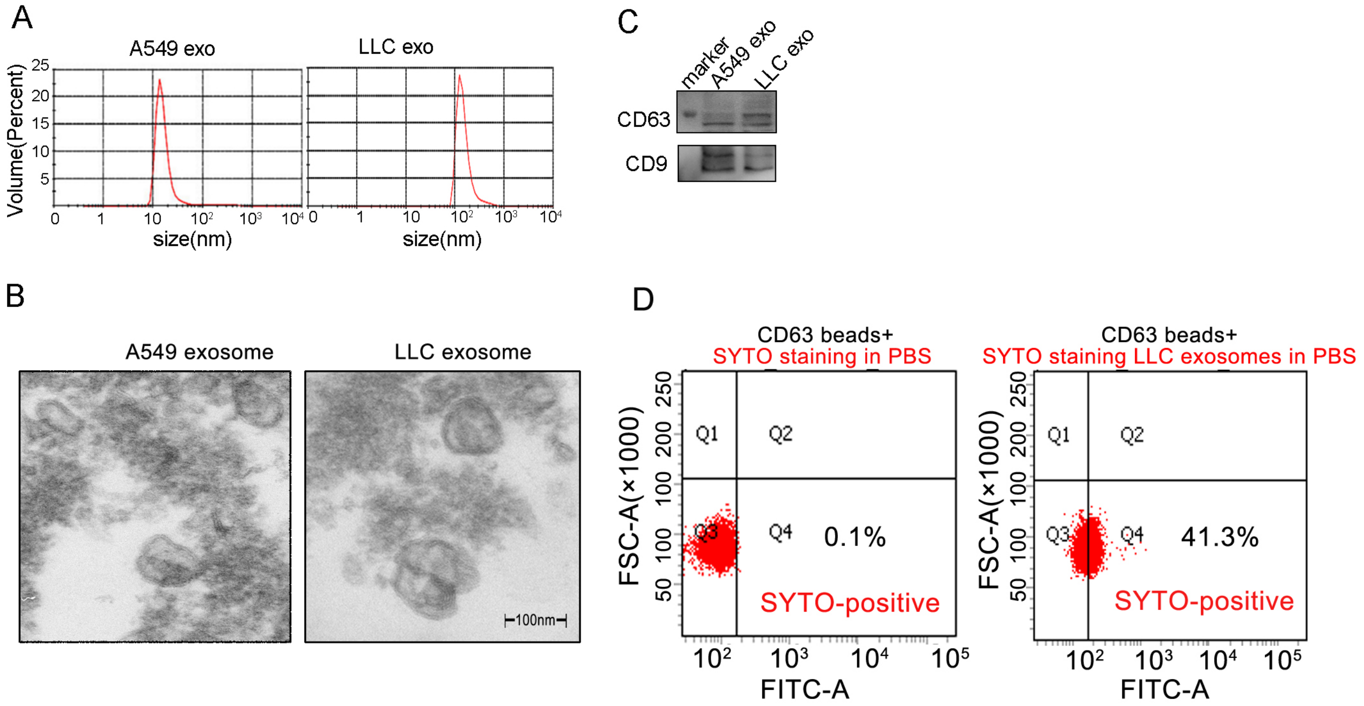

Supplementary Fig.2

A

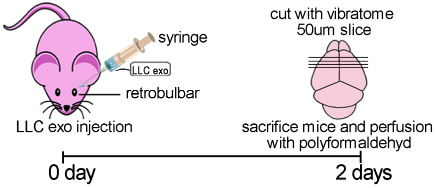

B

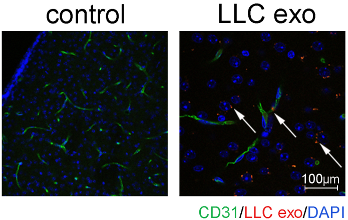

Supplementary Fig.3

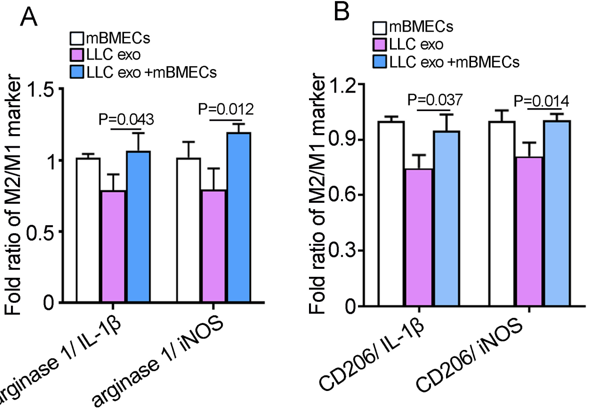

Supplementary Fig.4

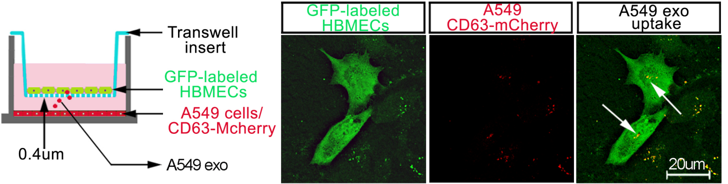

Supplementary Fig. 5

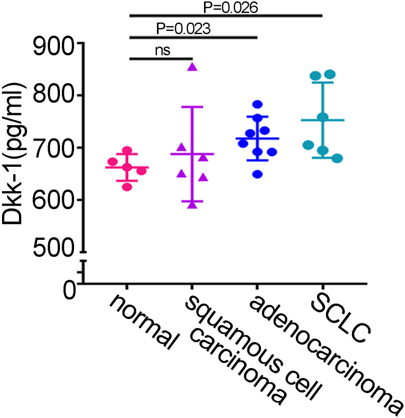

Supplementary Fig.6

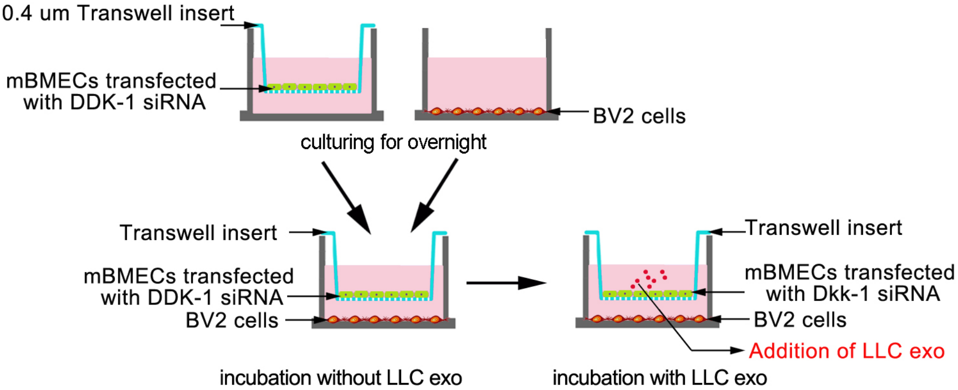

Supplementary Fig.7

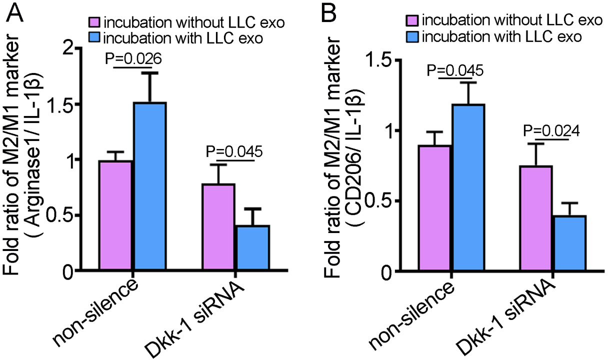

Supplementary Fig.8

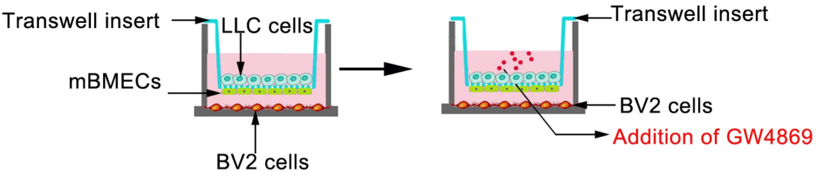

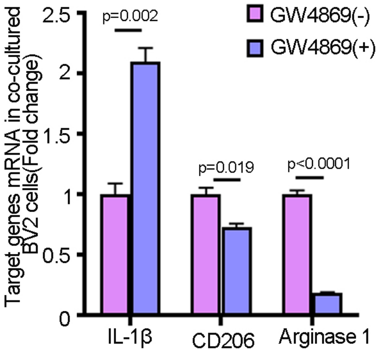

Supplementary Fig. 9

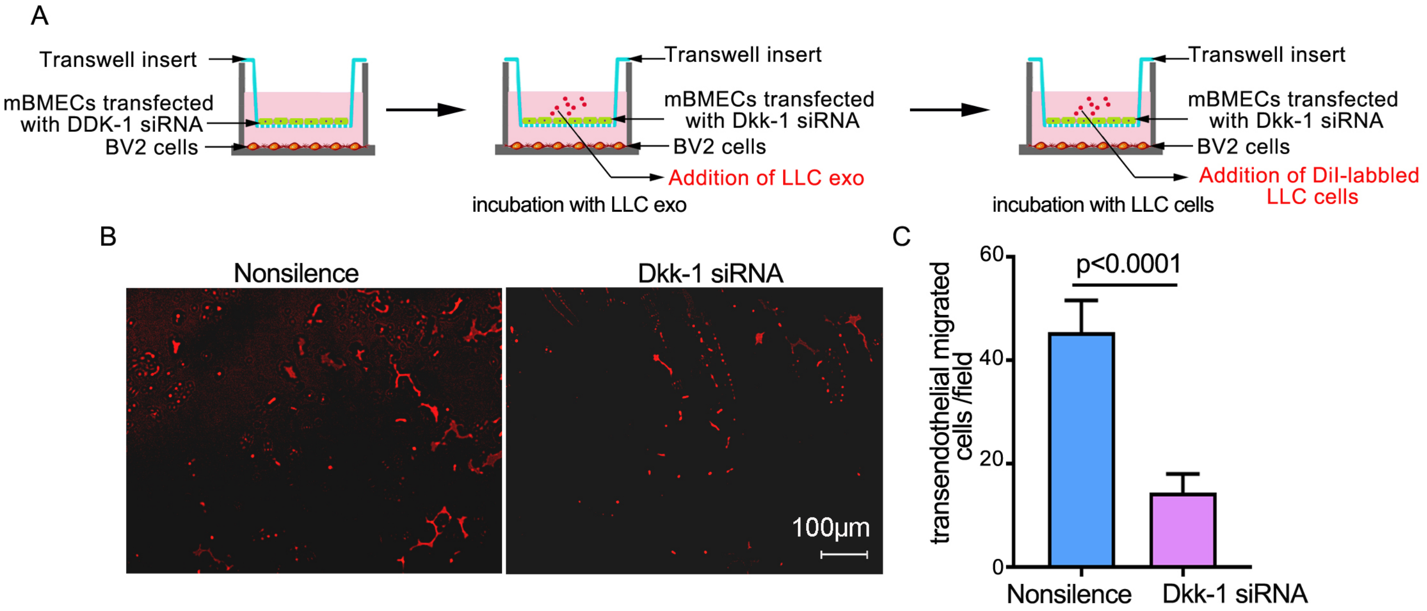

Supplementary Fig. 10

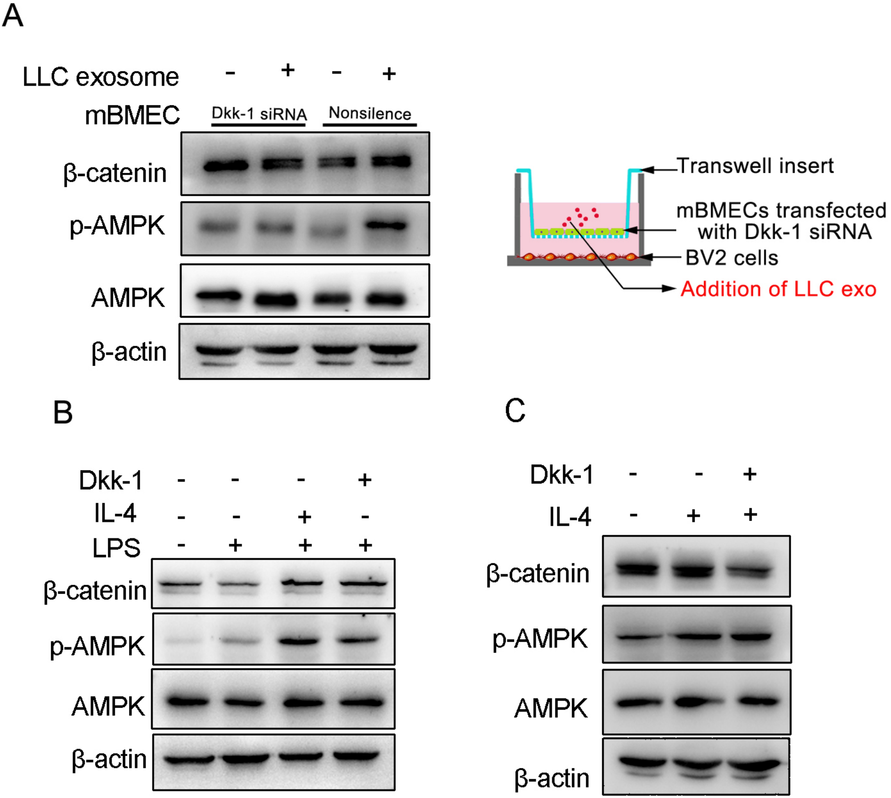

Supplementary Fig.11

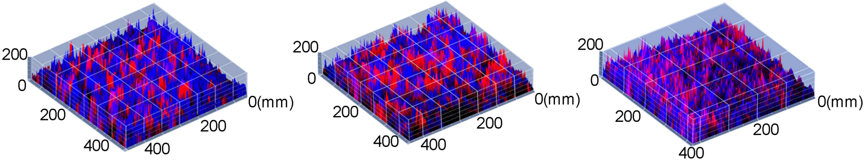

LLC-BrM1 LLC-BrM2 LLC-BrM3

Supplementary Fig.12

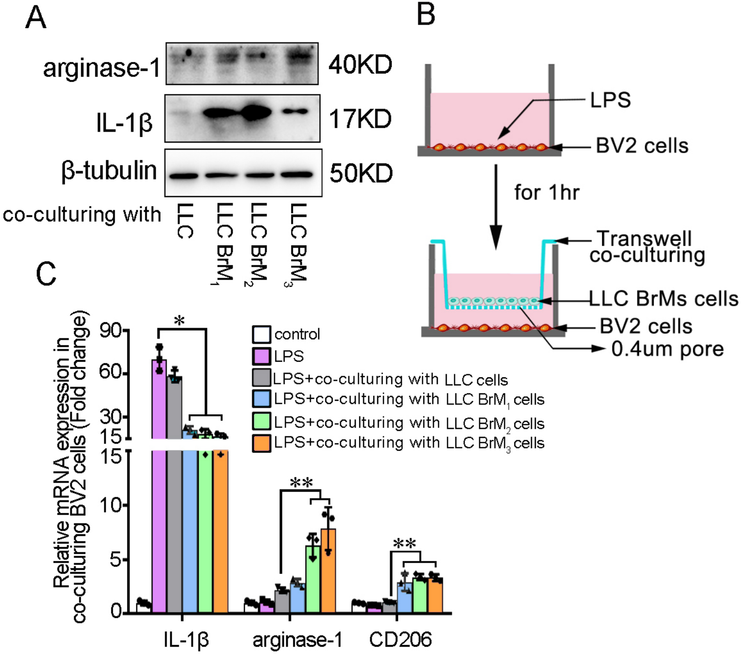

Supplementary Fig. 13

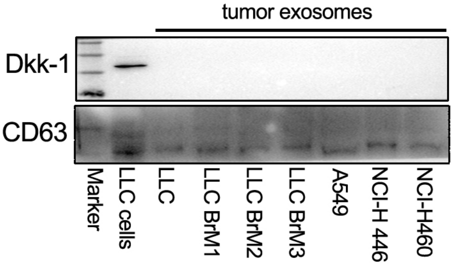

Supplement: Supplementary file 1 [file Data_Sheet_1.PDF]
